# Comprehensive multiplexed protein quantitation delineates eosinophilic and neutrophilic experimental asthma

**DOI:** 10.1186/1471-2466-14-110

**Published:** 2014-07-04

**Authors:** Maria Bergquist, Sofia Jonasson, Josephine Hjoberg, Göran Hedenstierna, Jörg Hanrieder

**Affiliations:** 1 The Hedenstierna Laboratory, Department of Medical Sciences, Uppsala University, Uppsala, Sweden; 2 Swedish Defence Research Agency, Division of CBRN Defence and Security, Umeå, Sweden; 3 Respiratory & Inflammation Innovative Medicines, AstraZeneca R&D, Mölndal, Sweden; 4 Department of Chemical and Biological Engineering, Chalmers University of Technology, Kemivägen 10, Gothenburg, Sweden

**Keywords:** Asthma, Bronchoalveolar lavage, Endotoxin, Inflammation, Ovalbumin, Proteomics, Mass spectrometry

## Abstract

**Background:**

Improvements in asthma diagnosis and management require deeper understanding of the heterogeneity of the complex airway inflammation. We hypothesise that differences in the two major inflammatory phenotypes of asthma; eosinophilic and neutrophilic asthma, will be reflected in the lung protein expression profile of murine asthma models and can be delineated using proteomics of bronchoalveolar lavage (BAL).

**Methods:**

BAL from mice challenged with ovalbumin (OVA/OVA) alone (standard model of asthma, here considered eosinophilic) or OVA in combination with endotoxin (OVA/LPS, model of neutrophilic asthma) was analysed using liquid chromatography coupled to high resolution mass spectrometry, and compared with steroid-treated animals and healthy controls. In addition, conventional inflammatory markers were analysed using multiplexed ELISA (Bio-Plex™ assay). Multivariate statistics was performed on integrative proteomic fingerprints using principal component analysis. Proteomic data were complemented with lung mechanics and BAL cell counts.

**Results:**

Several of the analysed proteins displayed significant differences between the controls and either or both of the two models reflecting eosinophilic and neutrophilic asthma. Most of the proteins found with mass spectrometry analysis displayed a considerable increase in neutrophilic asthma compared with the other groups. Conversely, the larger number of the inflammatory markers analysed with Bio-Plex™ analysis were found to be increased in the eosinophilic model. In addition, major inflammation markers were correlated to peripheral airway closure, while commonly used asthma biomarkers only reflect central inflammation.

**Conclusion:**

Our data suggest that the commercial markers we are currently relying on to diagnose asthma subtypes are not giving us comprehensive or specific enough information. The analysed protein profiles allowed to discriminate the two models and may add useful information for characterization of different asthma phenotypes.

## Background

Asthma is a heterogeneous airway inflammation which gives rise to several different clinical phenotypes. The phenotypes are traditionally classified according to their inflammatory profiles; eosinophilic asthma (EA), neutrophilic asthma (NA), mixed granulocytic asthma (MGA) and paucigranulocytic asthma (PGA) [1]. However, the disease relevant biochemistry underlying the differentiation of phenotypes remain unexplained and further research in the area could aid diagnosis accuracy and advance treatment.

Murine asthma models have been developed to mimic the two major subtypes of asthma, EA and NA. This has been achieved by intraperitoneal injections of ovalbumin (OVA) followed by either nebulization of OVA alone into the airways resembling the EA subtype, or adding nebulised endotoxin (lipopolysaccharide, LPS) together with OVA to create a neutrophilic airway inflammation [2-4]. The additional LPS exposure reflects a more severe form of experimental asthma, as it enhances the number of cells in bronchoalveolar lavage (BAL) and increases neutrophil recruitment, whereas the number of eosinophils have been reported to be both increased [2] and reduced [3]. Longitudinal in-depth investigations of related clinical specimen, such as BAL and lung tissue, represent a promising strategy to further elucidate the molecular pathology of these two asthma phenotypes. While common biochemical techniques have been the standard approach in molecular analysis of clinical samples, more powerful methodological approaches are needed to delineate molecular signatures in such complex biological systems.

Mass spectrometry based proteomics allows comprehensive and sensitive profiling of the protein expression pattern in biological samples [5]. We hypothesised that the pathogenic mechanisms underlying these asthma models would be reflected in the protein pattern in BAL. To this end, we therefore employed an integrated approach combining mass spectrometry-based protein analysis together with screening of a multiplex array of inflammatory biomarkers, in BAL in experimental asthma.

## Methods

### Animals

Female BALB/c mice (Taconic M&B, Denmark) were used in this study. They were housed in plastic cages with absorbent bedding material and were maintained on a 12 h daylight cycle. Food and water were provided ad libitum. Their care and the experimental protocols were approved by the Regional Ethics Committee on Animal Experiments in Uppsala (C86/5 and C64/8). Mice were 6–7 weeks of age when the airway inflammation protocol started and 9–10 weeks when BAL was collected (n = 5-6 mice per group).

### Asthma models

**Figure 1:**
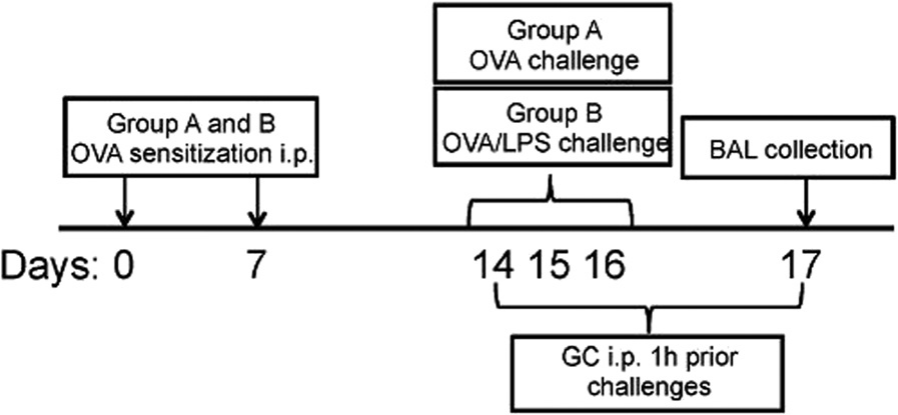
**Schematic outline of the animal experiments.** Two groups, resembling eosinophilic **(A)** and neutrophilic asthma **(B)**, were subjected to sensitization via i.p. injection and challenge through inhalation of ovalbumin (OVA). For the neutrophilic asthma model, animals were additionally challenged with lipopolysaccharide (LPS). A third group of animals in the neutrophilic asthma group, received steroid (GC) treatment 1 h prior challenge and lung mechanic assessment. As controls a final fourth group, received only vehicle (PBS) treatment during inhalation. Lung function testing was performed for all groups at day 17 followed by BAL fluid collection, differential cell count and proteomic analysis.

For the eosinophilic asthma group (OVA, n = 5), airway inflammation was induced as described previously [3] by intraperitoneal (i.p.) injections of 10 μg ovalbumin (OVA, Sigma-Aldrich, St. Louis, MO, USA) emulsified in Al(OH)_3_ (Sigma) on day 0 and day 7. The animals were then inhaled aerosolised 1% OVA diluted in phosphate-buffered saline (PBS, Sigma) for 30 min on days 14–16 (Figure 1). The aerosol exposure was performed in a chamber coupled to a nebuliser (DeVilbiss UltraNeb®, Sunrise Medical Ltd, U.K.). The chamber was divided into pie-shaped compartments with individual boxes for each animal, providing equal and simultaneous exposure to the allergen. A second group of mice (n = 6), resembling neutrophilic asthma, received i.p. OVA and was exposed to inhaled aerosolised LPS (*Escherichia coli* serotype 0111:B4; Sigma) dissolved in ddH_2_O) diluted in PBS simultaneously with OVA as described above on days 14–16 (Figure 1). The concentration of LPS in the nebuliser was 0.005% w/v (the OVA + LPS group). A third group (n = 5) received glucocorticoid (GC) treatment (hydrocortisone sodium succinate, 0.375 g/kg) immediately before OVA + LPS challenge (days 14–16). Finally, a group of mice (n = 5) served as control (C) with no exposure to any known airway irritant and was treated with vehicle (PBS).

### Lung mechanics and airway responsiveness

Dynamic lung mechanics were evaluated as described in detail elsewhere [3]. Briefly, airway reactivity was characterised by murine ventilator and forced oscillation technique (FOT) where Newtonian resistance (R_N_), tissue damping (G) and elastance (H) were determined. Airway responsiveness was determined by investigating the maximal response of G, H, an R_N_ upon intravenous methacholine (MCh) injection in incremental doses (0 (PBS), 0.03, 0.1, 0.3, 1, and 3 mg/kg). MCh (acetyl-β-methylcholine chloride, Sigma Aldrich) was diluted in PBS (Sigma Aldrich) with 10U/mL heparin. The volume of MCh solution was adjusted to 2 mL/kg that were injected for each dose.

### BAL collection and cell count

**Figure 2:**
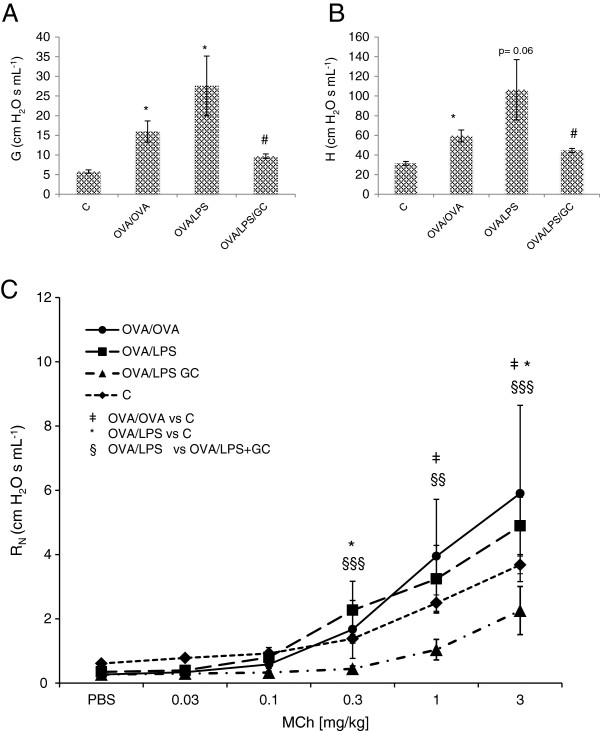
**Lung Mechanics: Airway responsiveness was evaluated using forced oscillation technique (FOT) [Prime 2 perturbation, resp. system impedance (Z**_**rs**_**) measurements]. (A,B)** Measurements of methacholine (MCh) induced tissue damping (G, A) and elastance (H, B). The maximum MCh response (3 mg/kg) was measured in controls (PBS), OVA/OVA challenged group, OVA/LPS challenged group and OVA/LPS challenged mice that received steroid treatment (OVA/LPS/GC). Values are indicated as mean ± SE.*p < 0.05 (C vs OVA/OVA and C vs OVA/LPS); #p < 0.05 (OVA/LPS vs OVA/LPS/GC); **(B)** p = 0.06 (C vs OVA/LPS); **(C)** Measurements of methacholine (MCh) induced Newtonian resistance (R_N_) for different MCh doses (mg/kg). Values are indicated as mean ± SE. ǂ,*,§: p < 0.05; §§: p < 0.01 (C vs OVA/LPS); §§§: p < 0.001 (C vs OVA/LPS); The control data have been published previously [4].

Mice were subjected to BAL via the tracheal tube (0.6 mM EDTA/PBS). BAL fluid was centrifuged, the cell pellet subjected to erythrolysis followed by cell count and cytospin preparations (50 000 cells, Shandon Cytospin 3) stained with May-Grünwald-Giemsa reagent. Differential cell counts of pulmonary inflammatory cells were made with standard morphological criteria counting 300 cells per cytospin preparation (Figure 2).

### Proteomic profiling

#### Protein digestion

The total protein concentration in the different BAL samples was determined using a Bradford assay (Protein Assay, BioRad, Hercules, MA). The samples were normalised to a total protein amount of 50 μg. A volume of 50 μL denaturation buffer (8 M urea, 400 mM NH_4_HCO_3_, Sigma) was added, followed by the addition of 10 μL DTT (45 mM, Sigma) and incubation at 55°C for 15 min for protein reduction. For alkylation a volume of 10 μL IAA (100 mM, Sigma) was added followed by incubation at 25°C in darkness. 25 μg sequence grade trypsin from bovine pancreas (Roche, Basel, Switzerland) were reconstituted in 250 μL ddH_2_O to give a final concentration of 100 ng/μL. A volume of 20 μL Trypsin solution (2 μg, 1:25 w:w) was added to the protein solution and incubated at 37°C overnight. The samples were desalted on ZipTip® C18 columns (Millipore, Bedford, MA, USA), according to manufactures instructions. The collected peptide fractions were dried down under reduced pressure (Thermo Savant SpeedVac, Thermo Scientific, Hercules, MA, USA) and reconstituted in 10 μL 0.1% formic acid (FA).

#### LC –ESI MS/MS

The tryptic peptide samples were analysed in duplicates on an Agilent 1100 nanoflow system (Agilent Technologies, Santa Clara, CA, USA) hyphenated to a LTQ-FT 7.0 T electrospray linear iontrap/Fourier transform ion cyclotron resonance MS hybrid instrument (Thermo). A volume of 5 μL from the reconstituted digests was injected automatically and loaded onto a in-house packed C18 PicoFrit column (75 μm ID/15 μm tip ID, NewObjective, Woburn, MA, USA) packed directly inside the electrospray needle tip using specially designed nanospray emitter tips. A water/formic acid/acetonitrile solvent system was used where solvent A was 0.1% FA and solvent B was 100% ACN, 0.1% FA. Gradient elution was performed from 0% B for 10 min, then from 0% B to 50% B for 100 min, then from 50% B to 90% B for 5 min, then 90% B for 5 min and finally from 90% B back to 0% B for 5 min. Peptide elution was followed by ESI FTICR MS and tandem mass spectrometry (MS/MS) for peptide sequencing controlled by the Xcalibur software (v.2.0 SR2, Thermo). Fullscan spectra were acquired at high resolution (FWHM = 100000) using the FT analyser. Data dependent acquisition was applied for MS/MS precursor selection, where the 5 most intense mass peaks were subjected to subsequent isolation and collision-induced fragmentation in the ion trap. Acquired raw data were exported to an *.mgf file using an in-house written script (C++). The annotated fragment spectra were subjected to database search using, the Mascot search engine (v.2.2, Matrix Science, London, UK) (5). Mascot searches were performed against the Uniprot knowledgebase (v.56, http://www.uniprot.org) using the following specifications: mass tolerance (MS: ±10 ppm, MS/MS: ±0.9 Da) enzyme (trypsin), fixed modifications (carbamidomethyl), variable modifications (oxidation of Met), precursor charge (1+,2+,3+) and instrument (ESI-TRAP). Peptide matches with a score above the confidence threshold (p < 0.05) were considered to be a significant hit. A minimum number of 2 peptides per proteins were required. The false positive identification rate (FPR) was estimated by searching the data against a decoy database. Database searches were refined by narrowing the mass tolerance and only protein findings at a FPR <1% were considered.

#### Protein quantification

The database search results were exported as *.dat files and loaded into the Scaffold software (v.3.1.2, Proteome Software, Portland, OR) together with the corresponding protein sequence data file of the current uniprot database (v.56, *.fasta file, taxonomy: mouse; http://www.uniprote.org). Quantification was performed according to the normalised spectral count of each protein species (SI_N_) [5]. Relative protein intensities in each biological replicate were subjected to global statistical analysis (ANOVA, p < 0.05) to reveal significant differences in between the different groups using the corresponding function implemented in the software. The quantitation results were exported to MS Excel (v.2010) for further statistical evaluation.

### Multiplexed ELISA analysis

Inflammatory mediators in BAL were analysed for the presence of 23 cytokines and chemokines (Bio-Plex™ Pro Mouse Cytokine 23-plex panel, BioRad, Hercules, CA, USA) (Table 1). The analysis was performed in duplicates on a Bio-Plex™ system (Luminex Bio-Plex™ 200 System, Bio-Rad) according to the manufacturer’s instructions.

**Table 1:** Overview of protein species identified with quantitative proteomics that displayed significant changes in between different groups

**Protein species**	**Database annotation**^**1**^
Protein S100-A9	S10A9_MOUSE
Complement Factor B	CFAB_MOUSE
Phosphoglycerate mutase 1	PGAM1_MOUSE
Regenerating islet-derived protein 3-gamma	REG3G_MOUSE
Plasminogen	PLMN_MOUSE
Ig gamma-1 chain C, membrane-bound form	IGH1M_MOUSE
Pulmonary surfactant-associated protein	SFTPD_MOUSE
Plastin 2	PLSL_MOUSE
Polymeric immunoglobulin receptor	PIGR_MOUSE
C-X-C motif chemokine 15	CXL15_MOUSE
Tubulin polymerization-promoting protein 3	TPPP3_MOUSE
Copper transport protein ATOX1	ATOX1_MOUSE
Ceruloplasmin	CERU_MOUSE
Histone H2B type 1-A	H2B1A_MOUSE
Immunoglobulin J chain	IGJ_MOUSE
Serum albumin	ALBU_MOUSE
Serine protease inhibitor A3K	SPA3K_MOUSE
Eosinophil cationic protein 2	ECP2_MOUSE
Complement C3	CO3_MOUSE
Chitinase-3-like protein 3	CH3L3_MOUSE
Fibronectin	FINC_MOUSE
Resistin-like alpha	RETNA_MOUSE
Malate dehydrogenase, cytoplasmic	MDHC_MOUSE
Serine protease inhibitor A3N	SPA3N_MOUSE
Cathelin-related antimicrobial peptide	CRAMP_MOUSE
Glutathione reductase, mitochondrial	GSHR_MOUSE
Peptidoglycan recognition protein 1	PGRP1_MOUSE
Glyceraldehyde-3-phosphate dehydrogenase	G3P_MOUSE
Carbonyl reductase [NADPH] 2	CBR2_MOUSE
Histone H4	H4_MOUSE
14-3-3 protein epsilon	1433E_MOUSE

Proteins significantly identified by mass spectrometry based proteomics (p < 0.05) that were found significantly changed (p < 0.05, ANOVA) in between at least 2 groups. ^1^Protein annotation according to the uniprot knowledgebase (v.56, http://www.uniprot.org).

### Data analysis and statistics

For proteins that exhibited changes in concentration as revealed by label free quantitative proteomics, intensity values were pooled with Bio-Plex™ protein concentration data. The protein concentration data were mean centred and autoscaled prior subjection to principal component analysis using the *pcamethods* script (http://www.bioconductor.org) in R (http://www.R-project.org). For all individual protein species, ANOVA was performed followed by Tukey posthoc analysis (origin v.8.1, originlab, Northampton, MA, USA).

## Results

### Characterization of the experimental asthma models

For characterization of lung mechanics and airway reactivity, a murine ventilator and forced oscillation technique (FOT) was employed. This approach allowed to calculate respiratory system input impedance that in turn allows the lung mechanics to be divided into central and peripheral components as described previously [3,6]. This included Newtonian resistance (R_N_) as primary central parameter; and tissue damping (G) and elastance (H) as peripheral parameters (Figure 2) [3,6].

At maximum dose MCh (3 mg/kg), tissue damping (G) was increased in both OVA/OVA and OVA/LPS compared to controls (p < 0.05). Tissue damping was increased in OVA/OVA compared to OVA/LPS, although not significant (p = 0.07). Steroid treatment (OVA/LPS/GC) reduced G (p < 0.01) as compared to the OVA/LPS group (Figure 2A). Upon MCh injection at maximum dose (3 mg/kg), elastance (H) was increased in OVA/OVA (p < 0.05) and OVA/LPS (p = 0.06) compared to control animals. H was furthermore significantly decreased (p < 0.05) upon GC treatment (OVA/LPS/GC) compared to OVA/LPS mice (Figure 2B). MCh induced bronchoconstriction (R_N_) was increased in both asthma models compared to controls (p < 0.05) for the maximum MCh dose. Similarly, R_N_ was significantly decreased with steroid treatment (Figure 2C). No significant changes were observed for MCh induced Newtonian resistance in between OVA/OVA and OVA/LPS mice.

**Figure 3:**
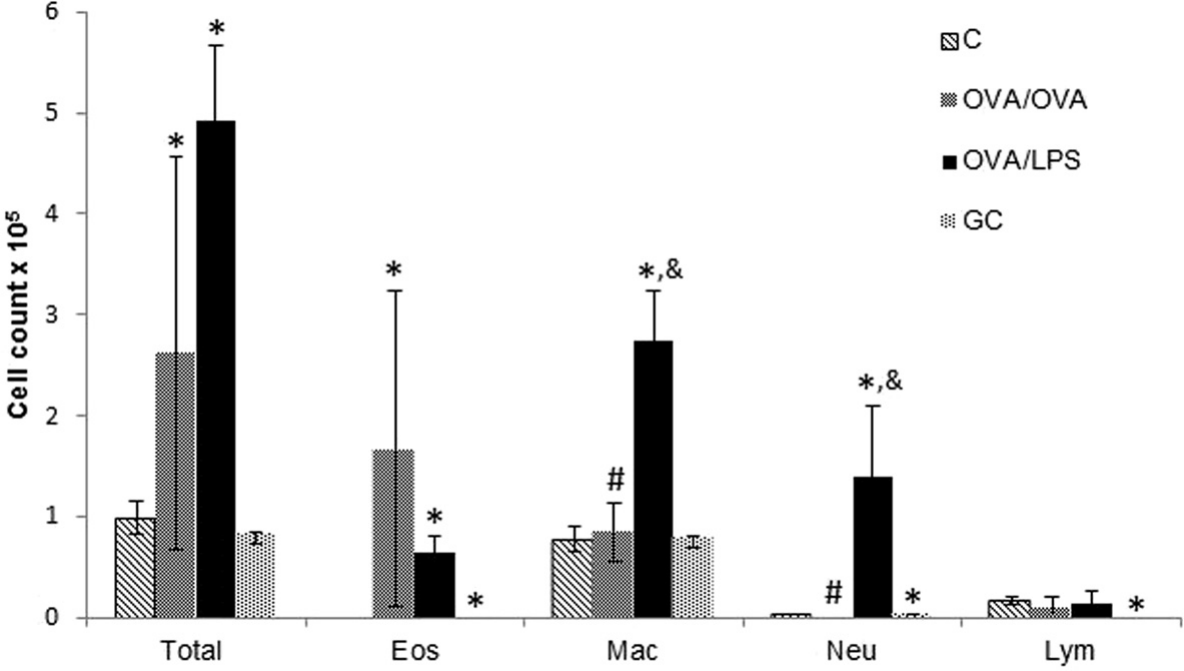
**Total cell count for inflammatory cells (mean ± SEM) including eosinphils (Eos), macrophages (Mac), neutrophils (Neu) and lymphocytes (Lym) for each treatment group.** Non-parametric ANOVA (Kuskal Wallis) revealed statistical significance between *Controls* (*C*) and *OVA/OVA* as well as *C* and *OVA/LPS* group for total cell counts, eosinophils, macrophages and neutrophils (*p < 0.05). For *C* vs *GC* significant difference was observed for lymphocytes (*p < 0.05). Significant difference between *OVA/LPS* and *GC* group was observed for macrophages and neutrophils (& p < 0.05) as well as a strong trend (p = 0.0504) for eosinophils. For macrophages and neutrophils significant difference were observed in between *OVA/OVA* and *OVA/LPS* (#p < 0.05). The control data have been published previously [4].

Lung mechanics were complemented with total BAL cell count for inflammatory cells including eosinphils (Eos), macrophages (Mac), neutrophils (Neu) and lymphocytes (Lym) for each treatment group. Here, a significant increase of total cell counts, eosinophils, macrophages and neutrophils was observed between control and OVA/OVA as well as C and OVA/LPS group for (p < 0.05).Furthermore, an increase of macrophage and neutrophil numbers (p < 0.05) was observed in OVA/LPS challenged mice compared to the OVA/OVA group. In addition, macrophages and neutrophil numbers were decreased in steroid treated mice (OVA/LPS/GC group) compared to OVA/LPS mice (p < 0.05) (Figure 3). Furthermore, eosinophil numbers were decreased in OVA/LPS/GC compared to OVA/LPS, although this was a strong trend (p = 0.0504), this decrease was not significant. Lymphocyte numbers did not display a change in between the different treatment groups.

### Differential BAL proteome profiling in experimental asthma

**Figure 4:**
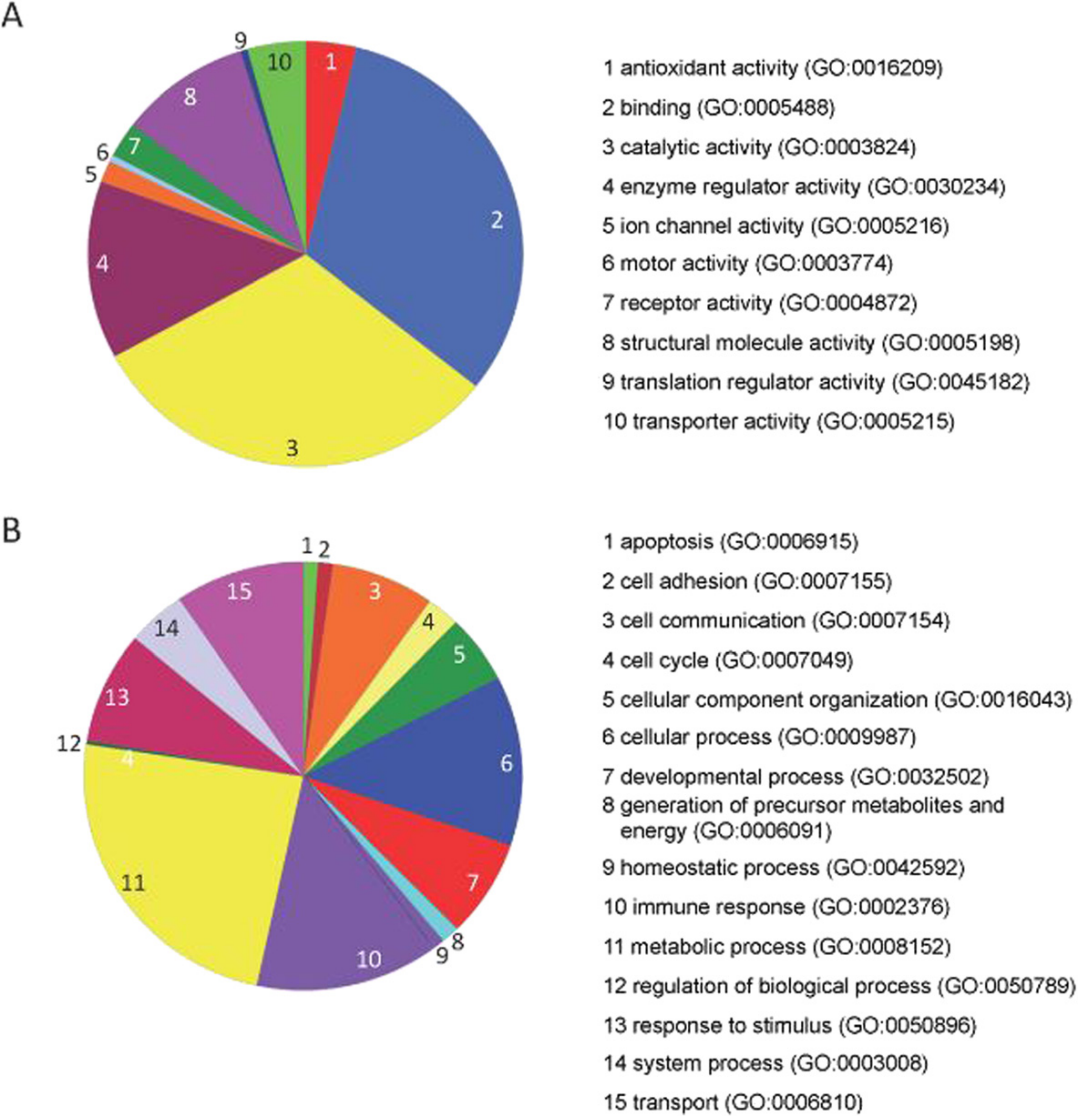
**Protein function and relevance in various biological processes as determined by PANTHER/Gene Ontology analysis. (A)** Gene ontology map of detected protein species: molecular function (read clockwise starting at 1 = red to 10 = green). **(B)** Gene ontology map of detected protein species: biological process (read clockwise starting at 1 = green to 15 = pink).

Comprehensive proteomic profiling of BAL using nanoLC-ESI FTICR MS/MS yielded 176 significant and unique protein species that were identified consistently in all 30 BAL samples (Additional file 1: Table S1). In order to determine protein functionalities, all proteomic data were mapped according to the individual molecular function and biological process using the PANTHER (Protein ANalysis THrough Evolutionary Relationships) Classification System [7], a part of the gene ontology project. A large part of the detected protein species were found to be involved in immune response (Figure 4B) as well as rather general processes such as cell communication, metabolism and transport (Figure 4A). In detail, the proteins had a wide variety of different functionalities, including binding, catalytic and enzymatic activity (Figure 4B).

Statistical analysis of the normalised spectral count data (SI_N_) of all identified protein species revealed significant changes in protein intensities between the different groups. Statistical analysis (ANOVA, Tukey posthoc) showed significant changes for 28 protein species (p < 0.05, Table 1, Additional file 2: Figure S1). Due to the dynamic concentration range, detection of chemokines using LC-MS based proteomics is difficult and requires targeted approaches such as ELISA. Therefore the aim was to complement the proteomic data with a standard panel of well-known chemokines that are of established relevance in airway inflammation. Here, complementary multiplexed ELISA (Bio-Plex™) analysis added information about common inflammatory markers in the groups (Table 2). Of the 23 measured chemokines, a number of 17 were significantly changed in between the different groups (p < 0.05; Additional file 2: Figure S2).

**Table 2:** **Overview of Protein species included in the Bio-Plex™panel for multiplexed ELISA**

**Protein name**	**Abbreviation**
Interleukin 1a	IL-1a*
Interleukin 1b	IL-1b*
Interleukin 2	IL-2*
Interleukin 3	IL-3*
Interleukin 4	IL-4
Interleukin 5	IL-5
Interleukin 6	IL-6
Interleukin 9	IL-9*
Interleukin 10	IL-10
Interleukin 12 p40	IL-12(p40)*
Interleukin 12 p70	IL-12(p70)
Interleukin 13	IL-13*
Interleukin 17	IL-17*
Eotaxin	Eotaxin*
Granulocyte colony-stimulating factor	G-CSF
Granulocyte-macrophage colony-stimulating factor	GM-CSF*
Interferon gamma	IFN-γ*
Chemokine (C-X-C motif) ligand 1	KC*
Monocyte chemotactic protein-1)	MCP-1*
Macrophage Inflammatory Protein 1a	MIP-1a*
Macrophage Inflammatory Protein 1b	MIP-1b*
Chemokine (C-C motif) ligand 5	RANTES*
Tumor necrosis factor alpha	TNFα*

*Marked species were significantly (p < 0.05) changed in between at least 2 groups.

### Multivariate data analysis of integrative proteomic fingerprints

**Figure 5:**
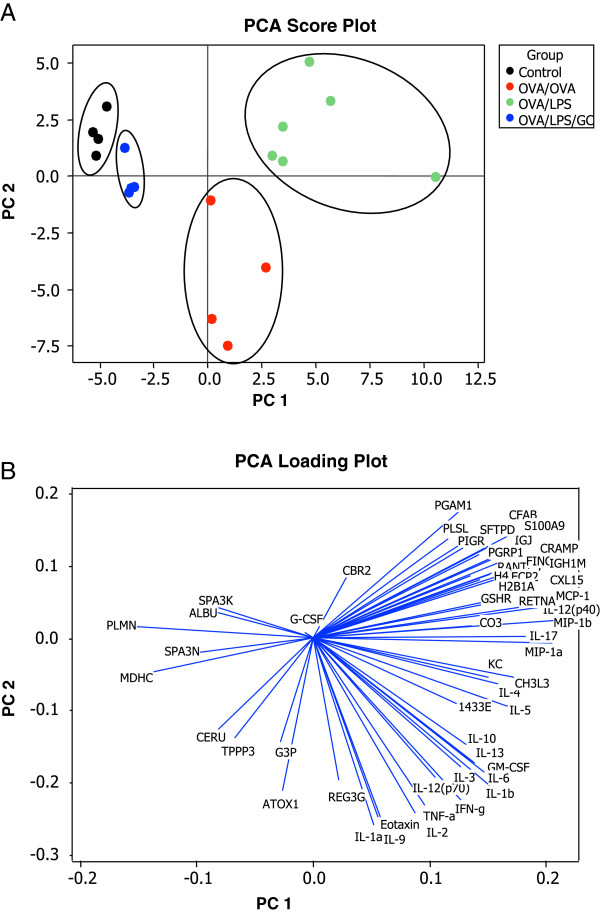
**Statistical discrimination of different experimental asthma models and glucocorticoid treatment.** Multivariate analysis by means of principal component analysis (PCA) of all group specific protein concentration levels allowed clear separation of the different samples according to their treatment group **(A)**. The corresponding loading plot shows the loading of each variable (protein concentration) on the score for each individual sample. This in turn allows deducing what protein species impact the sample scores and their clustering behaviour **(B)**.

For further data analysis by means of multivariate statistics, the proteomics data as well as the Bio-Plex™ data were combined in a single data matrix and subjected to principal component analysis (PCA). The results show distinct clustering of the individual samples according to their respective group (Figure 5A). Inspection of the corresponding loadings enabled for deduction of the individual variables (protein intensities) that had the greatest influence on the corresponding PC score for each individual sample. The PC score based clustering behaviour is reflected in the corresponding loadings and therefore based on similar changes of the protein intensities that relate to these loadings (Figure 5B). This reveals the individual protein species that show similar changes based on different models and allow differentiation of the individual samples based on their multivariate pattern.

### Altered protein expression in different subtypes of experimental asthma and GC treatment

**Figure 6:**
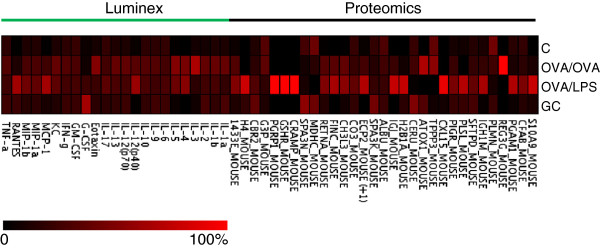
**Protein changes in different experimental models of asthma (OVA and OVA + LPS) as well as glucocorticoid treated animals (GC) and controls (C).** Heat map showing protein quantification results (mean SI_N_, n = 5-6) detected by MS based proteomics and Bio-Plex™. The data are normalised to the total intensity as indicated by the intensity scale.

Inspection of the variables (loadings, proteins) as obtained by multivariate analysis, revealed group specific protein regulation patterns (Figure 5B). These results were compared to univariate statistical analysis (ANOVA). Many proteins displayed significant differences between the controls and either or both of the two models reflecting EA and NA (Figure 6, Additional file 2: Figure S1 and S2). The major number of proteins were found to be only slightly or not at all increased in EA (OVA) compared to controls, but displayed a prominent increase in NA (OVA + LPS-induced) compared to all other groups (Figure 6). These included mainly acute phase reactants, such as S100 calcium binding protein A9 (calgranulin B/S100-A9), complement CO3 (CO3), complement factor B (CFAB), immunoglobulins IG-J and IG-H as well as histones (H2 and H4) and phosphoglycerate mutase (PGAM1). Furthermore, similar trends were observed for proteins of potential relevance in the respiratory system, including eosinophil cationic protein (ECP2), lung polymeric immunoglobulin receptor (PIGR) and pulmonary surfactant protein D (SFTPD) (Additional file 2: Figure S1). Pro-inflammatory markers Monocyte Chemotactic Protein 1 (MCP1) and Regulated upon activation normal T cell expressed and presumably secreted (RANTES) detected in the Bio-Plex™ analysis panel showed a marked elevation in the LPS group (Additional file 2: Figure S2).

A number of protein species were found increased in both asthma models. Eosinophil cationic protein 2 (ECP2), resistin A (RETNA), fibronectin (FINC) and chitinase 3 (CH3L3) exhibited a higher intensity in the NA compared to the EA model, but were increased in EA compared to controls and glucocorticoid-treated animals (Additional file 2: Figure S1). The same trend was found for MIP-1α and β, as well as interleukins IL-4, IL-12p40, and IL-17A. Conversely, IL-1β, IL-2, IL-5, IL-10 and keratinocyte chemo-attractant (KC) were elevated in both models but higher in EA compared to NA (Additional file 2: Figure S2). Finally, five protein species including regenerating islet-derived protein 3 (REG3), tubulin polymerization promoting protein (TPPP), IL-3, eotaxin and interferon gamma (IFN-γ) were found solely elevated in the EA group and not in the NA group (Additional file 2: Figure S1 and S2). Proteins found in control mice that were negatively regulated by airway inflammation and recovered after glucocorticoid treatment was malate dehydrogenase (MDHC) and serine protease inhibitor 3 (SPA3N). Plasminogen (PLMN) was decreased both in the EA and the NA groups, but was not recovered by steroid treatment (Figure 6, Additional file 2: Figure S1 and S2).

### Correlation between specific proteins and inflammatory cells

Linear regression analysis was performed for all significant protein species and the total cell count for inflammatory leucocytes (Table 3). Here, positive correlations were observed for the neutrophil count with acute phase reactants (S100-A9), immunoglobulins (IGH1M, PIGR), metabolic enzymes (PGAM) as well as other multifunctional proteins including actin-binding protein plastin 2 (PLSL), fibronectin (FINC), CRAMP and PGRP1. Eosinophils were found to correlate positively with cytokines IL-9 and IFN-γ, as well as eotaxin and carbonyl reductase 2 (CBR2). Lymphocyte count correlated positively with IGHM1, PIGR and FINC, but interestingly, was found to correlate negatively with CBR2. Macrophage count displayed positive correlations with S100-A9, CFAB, cytokines (IL-12p40, IL-13, GM-CSF, MIP-1b, TNF), chemokines (CXCL-15, CH3L3), binding proteins (PLSL, H2B1A and H4), immunoglobulins (IGJ, IGH1M), cytotoxic protein ECP2, adaptor protein 1433e, peptidoglycan recognition protein PGRP1, antimicrobial peptide CRAMP, and mitochondrial protein GSHR. The anticoagulant and proteolytic factor plasminogen (PLMN) displayed a negative correlation with macrophages.

**Table 3:** Pearson correlation coefficients of BAL protein levels versus inflammatory cell counts

	**TOTAL**	**NEU**	**EOS**	**MAC**	**LYM**
S100-A9	0.732	0.898	ns	0.698	ns
CFAB	ns	ns	ns	0.811	ns
PGAM1	ns	0.930	ns	ns	ns
PLMN	-0.756	ns	ns	-0.884	ns
IGH1M	0.867	0.894	ns	0.656	0.717
PLSL	ns	0.780	ns	0.621	ns
PIGR	ns	0.736	ns	ns	0.704
CXCL-15	ns	ns	ns	0.876	ns
H2B1A	ns	ns	ns	0.710	ns
IGJ	ns	ns	ns	0.732	ns
ECP2	ns	ns	ns	0.655	ns
CH3L3	0.807	ns	ns	0.787	ns
FINC	0.676	0.824	ns	ns	0.743
CRAMP	0.664	0.755	ns	0.767	ns
GSHR	ns	ns	ns	0.868	ns
PGRP1	ns	0.643	ns	0.769	ns
H4	ns	ns	ns	0.885	ns
1433E	ns	ns	ns	0.663	ns
CBR2	ns	ns	0.818	ns	-0.755
CO3	0.736	ns	ns	ns	ns
IL-2	ns	ns	0.682	ns	ns
IL-9	ns	ns	0.788	ns	ns
IL-12p40	ns	ns	ns	0.716	ns
IL-13	0.771	ns	ns	0.860	ns
Eotaxin	0.688	ns	0.723	ns	ns
Interferon γ	ns	ns	0.663	ns	ns
GM-CSF	0.758	ns	ns	0.650	ns
MIP-1b	ns	ns	ns	0.688	ns
TNF	ns	ns	ns	0.679	ns

*Definition of abbreviations*: *Neu* neutrophils, *Eos* eosinophils, *Mac* macrophages and *Lym* lymphocytes. R > 0.5 and R < -0.5 are considered strong positive and negative linear correlation, respectively. Ns: no significant correlation, p > 0.05.

### Correlation between specific proteins and lung mechanics

Correlation analysis of individual protein concentration values obtained from both proteomic strategies (LCMS and Bioplex) and lung mechanics data were performed for each animal. Here, peripheral lung mechanics parameters; elastance (H) and tissue damping (G) were found to correlate positively with inflammatory markers (S100-A9, RANTES), immunoglobulins (IGH1M, PIGR), metabolic enzymes (PGAM) as well as other functional proteins including actin-binding protein plastin 2 (PLSL), fibronectin (FINC), CRAMP, PGRP1 (only G) and interleukins: IL12p40 and IL17 (only G) (Table 4). Newtonian resistance (R_N_) as central lung mechanic parameter was found to correlate negatively with serine protease inhibitor (SPA3K) and carbonyl reductase (CBR2). Conversely, positive correlations were obtained in between R_N_ and chitinase (CH3L3) as well as interleukins: IL5, IL12p40 and IL13 (Table 4).

**Table 4:** Pearson correlation coefficients of BAL protein levels versus lung mechanics

	**G**	**H**	**R**_**N**_
S100-A9	**0.85**	**0.8**	ns
PGAM1	**0.92**	**0.88**	ns
IGH1M	**0.91**	**0.87**	ns
PLSL	**0.74**	**0.75**	ns
PIGR	**0.75**	0.71	ns
SPA3K	ns	ns	-0.64
CH3L3	ns	ns	0.69
FINC	**0.86**	**0.83**	ns
CRAMP	0.67	0.59	ns
PGRP1	0.56	ns	ns
MDHC	ns	ns	ns
CBR2	ns	ns	-0.71
IL 5	ns	ns	0.6
IL 12p40	0.65	0.59	0.6
IL 13	ns	ns	**0.93**
IL 17	0.57	ns	ns
RANTES	0.7	0.67	ns

Methacholine induced (3 mg/kg) tissue damping (G); tissue elastance (H) and Newtonian resistance (R_N_). Regression coefficients R > 0.5 and R < -0.5 are considered strong positive and negative linear correlation, respectively. ns: no significant correlation, p > 0.05. Bold values indicate significant correlation with p < 0.01.

## Discussion

Over the last decade, proteomic based mapping of the protein expression profiles of complex biological samples has been well established for getting a comprehensive molecular insight in biological processes underlying disease pathology. To our knowledge, this is the first study on quantitative proteomic profiling of lung-derived specimen in experimental eosinophilic and neutrophilic asthma. We hypothesised that protein expression patterns of BAL fluid would reflect the mechanistic differences between asthma phenotypes. In this study, we therefore investigated BAL proteome dynamics from experimental eosinophilic and neutrophilic asthma using an integrated proteomics approach based on high resolution mass spectrometry and multiplexed ELISA.

We demonstrated that the protein expression levels of several acute phase proteins such as S100-A9, complements (CO3, CFAB) and immunoglobulins (IGJ, IGH, PIGR) were increased in the BAL from mice with OVA + LPS-induced airway inflammation compared to mice with OVA-induced airway inflammation, and that these up regulations could be nearly completely averted by pre-treatment with glucocorticoid therapy (Additional file 2: Figure S1 and S2). Our major findings show that the eosinophilic (OVA-induced) and the neutrophilic (OVA + LPS induced) asthma models encompass significant and relevant differences in their protein patterns, which could not be delineated by common techniques used for characterization of airway inflammation, such as inflammatory cell counts and lung mechanics (Figures 2 and 3). Using multivariate data analysis allowed for discriminating the two asthma models from each other, as well as from healthy control and steroid treated animals (Figures 5).

The most characteristic protein regulations associated with neutrophilic experimental asthma included increased levels of acute phase reactants. The adaptive immune response is traditionally expected to be steroid sensitive, while the innate is expected to be steroid resistant [7]. The Th17 driven response has been suggested to play a critical role for the innate host defence against bacteria in mammalian lungs through its ability to indirectly mobilise neutrophils [8]. In line with this, our findings show an increased production of IL-17 as a result of the accumulated neutrophils after bacterial endotoxin stimulation *in vivo*, as well as a considerable decrease of IL-17 after steroid treatment. T cells have been reported to release IL-17 after endotoxin exposure, but only in the presence of macrophages [9]. IL-17 is suggested to be the strongest recruiter of neutrophils in lung tissue. In agreement with this, neutrophils and macrophages were increased in BAL from the NA group compared to the EA group (p < 0.05), in our model (Figure 3). Moreover, neutrophils and macrophages displayed strong positive correlations with other proteins elevated in the NA model (Table 3). The NA model resembles severe human asthma more than the more conventional EA model in that it shows Th17 response related characteristics such as IL17 expression and neutrophil recruitment. However, as previously demonstrated for LPS induced IL17 expression, effects of the NA model used in this study were attenuated upon steroid treatment [10], which in turn highlights the difficulties in creating experimental models of severe steroid-resistent human asthma.

The EA group could be delineated from the NA group based on the protein species; including TPPP3, IL-3, IFN-γ and eotaxin, which were found significantly elevated in the EA group compared to the NA group. In asthma, it is known that reducing histone deacetylases (HDAC) increases asthmatic inflammation and that glucocorticoids down regulate the inflammatory response in turn by modulating HDAC activity [11]. TPPP3 has been described to inhibit HDAC [12], possibly regulating the immune reaction towards the steroid sensitive Th2 response. Similarly, IL-3 has been associated with atopic asthma and Th2 response [13-15].

IFN-γ is traditionally distinguished as an essential Th1 response cytokine, but has been described to have a dual role and even protective effects in other disease models [16]. In the present study, IFN-γ was significantly increased in the EA group compared to the NA group. In addition, IFN-γ as well as eotaxin correlated strongly with eosinophil count.

As expected, the EA group had a marked increase of eotaxin expression. Eotaxin selectively attracts allergic effector cells, eosinophils and basophils, due to the requirement of a high affinity receptor (CCR3) [17,18]. Its synthesis is stimulated in different cell types by IL-4, IL-5 and IL-13, which is released mainly by Th2 lymphocytes [19]. Interestingly, eosinophilic cationic protein 2 (ECP2) was more pronounced in BAL from the NA group. This protein is localised in the cytoplasmic granula of eosinophils, with the main function to selectively attract dendritic cells to the source of infection. In spite the low cell count of eosinophils in BAL from the NA group, our data provide evidence that eosinophils indeed are present, but in the case of NA instead of recruiting more eosinophils, rather regulating the inflammation away from a Th2 response. Group specific protein regulations are therefore suitable markers for delineating different immune response mechanisms in between these models.

Complementally lung mechanics parameters, including elastance (H), tissue damping (G) and newtonian resistance (R_N_), showed a significant increase in the asthma models compared to the control group. While this verifies the animal model, both lung mechanics as well as BAL counts that are commonly used for characterizing asthma phenotypes, did not allow delineating the asthma models. However, correlation of lung mechanic data with the protein regulations revealed differences in peripheral and central parameters of airway responsiveness (Table 4). Here, strong correlation of peripheral parameters, elastance and tissue damping, correlated strongly with proteins elevated in NA. These correlations were found to be very similar to protein correlations observed for neutrophil and macrophage cell counts. Indeed, direct correlation analysis revealed a strong positive correlation for G (R = 0.99) and H (R = 0.97) with recruited neutrophils but not for other BAL cells. Conversely, Newtonian resistance as a central parameter for airway responsiveness displayed no correlation with any inflammatory cell count. This supports the theory that lung mechanics in the peripheral airways plays an important role in asthma pathophysiology due to exaggerated airway closure [20]. Thus, protein species associated with the NA phenotype also reflected peripheral airway closure. If confirmed, these proteins could serve as biomarkers indicating inflammation of distal airways.

Moreover, R_N_ was found to correlate with chitinase 3, a common biomarker in asthma. Chitinase 3 did not differentiate the two models of inflammation, although it has been suggested to play a key role in Th2 driven inflammatory response [21]. Similarly, further Th2 associated proteins, IL-5 and IL-13, correlated positively with R_N_. This suggests that commonly used markers for asthma, including IL-13 and chitinase, do in fact only reflect central airway inflammation.

## Conclusion

We employed an integrative multi-modal proteomic approach based on LC-FTICR-MS and Bio-Plex™ analysis for quantitative protein profiling of BAL samples in murine models of eosinophilic and neutrophilic asthma. The results show significant changes in protein expression between eosinophilic and neutrophilic murine asthma groups. These protein species may help to characterise the different phenotypes as well as the predominant mechanisms involved, particularly with respect to different T-lymphocyte mediated mechanisms in respiratory inflammation. Furthermore, the observed group-specific proteomic fingerprints can be used to characterise the specific patterns of clinical presentation and may be useful for future diagnosis, prediction of clinical outcomes and treatment guidance. In summary, most of the conventional inflammatory markers measured by the commercial Bio-Plex™ technique were increased in BAL from the EA group. In contrast, most of the proteins we could detect and quantify with LC-FTICR-MS were more prominent in the NA group. In addition, major inflammation markers were correlated to peripheral airway closure, while commonly used asthma biomarkers only reflect central inflammation. Therefore, our data suggest that the commercial markers we are currently relying on to diagnose asthma subtypes are not giving us comprehensive or specific enough information.

## Abbreviations

BAL: Bronchoalveolar lavage; EA: Eosinophilic asthma; NA: Neutrophilic asthma; OVA: Ovalbumin; LPS: Lipopolysaccharide; GC: Glucocorticoid; LC: Liquid chromatography; ESI: Electrospray ionization; FT: Fourier transform; MS: Mass spectrometry.

## Competing interest

The authors declare that they have no competing interests.

## Authors’ contribution

MB and JHa conceived and designed the study. SJ and JHj designed the animal model together with GH. SJ acquired and interpreted animal data. MB and JHa performed analysis and interpretation of the protein data. MB and JHa wrote the manuscript;MB, SJ, JHj, GH and JHa revised the manuscript, read and approved the final version of the manuscript.

## Pre-publication history

The pre-publication history for this paper can be accessed here:

http://www.biomedcentral.com/1471-2466/14/110/prepub

## Supplementary Material

Additional file 1: Table S1 Protein identified in BAL using mass spectrometry based proteomics. All proteins were identified at 95% significance level with at least 2 peptides. Accession Uniprot knowledgebase v.56 http://www.uniprot.org. Click here for file

Additional file 2: Figure S1 Protein changes as detected by means of mass spectrometry based proteomics. Statistical significance (p < 0.05) is indicated with * OVA/LPS vs C; # OVA/LPS vs OVA/OVA; % OVA/LPS vs OVA/LPS/GC and &OVA/OVA vs C. **Figure S2.** Protein changes as detected by means of Bio-Plex analysis. Statistical significance (p < 0.05) is indicated with * OVA/LPS vs C; # OVA/LPS vs OVA/OVA; % OVA/LPS vs OVA/LPS/GC and &OVA/OVA vs C. Click here for file
