# Supplementary material for: Comprehensive multiplexed protein quantitation delineates eosinophilic and neutrophilic experimental asthma
Source: BMC Pulm Med. 2014 Jul 4;14:110. doi: 10.1186/1471-2466-14-110 (PMC4137270; doi:10.1186/1471-2466-14-110)
Supplement: Additional file 1: Table S1 — Protein identified in BAL using mass spectrometry based proteomics. All proteins were identified at 95% significance level with at least 2 peptides. Accession Uniprot knowledgebase v.56 http://www.uniprot.org. [file 1471-2466-14-110-S1.docx]

**SI Table 1. Protein identified in BAL using mass spectrometry based proteomics.**

| Accession |  | Name |
| --- | --- | --- |
| 1433B_MOUSE | | 14-3-3 protein beta/alpha |
| 1433E_MOUSE | | 14-3-3 protein eta |
| 1433F_MOUSE | | 14-3-3 protein gamma |
| 1433T_MOUSE | | 14-3-3 protein theta |
| 1433Z_MOUSE | | 14-3-3 protein zeta |
| 6PGD_MOUSE | | 6-phosphogluconate dehydrogenase, decarboxylating |
| A1AG1_MOUSE | | Alpha-1-acid glycoprotein 1 |
| A1AG2_MOUSE | | Alpha-1-acid glycoprotein 2 |
| A1AT_MUSCR | | Alpha-1-antiproteinase |
| A1AT1_MOUSE | | Alpha-1-antitrypsin 1-1 |
| A1AT2_MOUSE | | Alpha-1-antitrypsin 1-2 |
| A1AT3_MOUSE | | Alpha-1-antitrypsin 1-3 |
| A1AT4_MOUSE | | Alpha-1-antitrypsin 1-4 |
| A2AP_MOUSE | | Alpha-2-antiplasmin |
| A2M_MOUSE | | Alpha-2-macroglobulin |
| ACBP_MOUSE | | Acyl-CoA-binding protein |
| ACTB_MOUSE | | Actin, cytoplasmic 1 |
| ACTA_MOUSE | | Actin, aortic smooth muscle |
| ACTBL_MOUSE | | Beta-actin-like protein 2 |
| ACTS_MOUSE | | Actin, alpha skeletal muscle |
| AFAM_MOUSE | | Afamin |
| AGR2_MOUSE | | Anterior gradient protein 2 |
| AK1A1_MOUSE | | Alcohol dehydrogenase [NADP+] |
| ADIPO_MOUSE | | Adiponectin OS |
| AL1A7_MOUSE | | Aldehyde dehydrogenase, cytosolic |
| ALBU_MOUSE | | Serum albumin OS |
| ALDOA_MOUSE | | Fructose-bisphosphate aldolase A |
| ALDR_MOUSE | | Aldose reductase |
| AMBP_MOUSE | | Protein AMBP |
| AMY1_MOUSE | | Alpha-amylase 1 |
| ANGT_MOUSE | | Angiotensinogen |
| ANT3_MOUSE | | Antithrombin-III |
| ANX11_MOUSE | | Annexin A11 |
| ANXA1_MOUSE | | Annexin A1 |
| ANXA2_MOUSE | | Annexin A2 |
| ANXA3_MOUSE | | Annexin A3 |
| ANXA4_MOUSE | | Annexin A4 |
| ANXA5_MOUSE | | Annexin A5 |
| APOA1_MOUSE | | Apolipoprotein A-I |
| APOA2_MOUSE | | Apolipoprotein A-2 |
| APOA4_MOUSE | | Apolipoprotein A-4 |
| APOH_MOUSE | | Beta-2-glycoprotein 1 |
| ATPB_MOUSE | | ATP synthase subunit beta, mitochondrial |
| B2MG_MOUSE | | Beta-2-microglobulin |
| CAD23_MOUSE | | Cadherin-23 |
| CAH2_MOUSE | | Carbonic anhydrase 2 |
| CATB_MOUSE | | Cathepsin B |
| CATC_MOUSE | | Dipeptidyl peptidase 1 |
| CATD_MOUSE | | Cathepsin D |
| CBR1_MOUSE | | Carbonyl reductase [NADPH] 1 |
| CBR2_MOUSE | | Carbonyl reductase [NADPH] 2 |
| CD14_MOUSE | | Monocyte differentiation antigen CD14 |
| CERU_MOUSE | | Ceruloplasmin |
| CES3_MOUSE | | Carboxylesterase 3 |
| CFAB_MOUSE | | Complement factor B |
| CFAH_MOUSE | | Complement factor H |
| CFAI_MOUSE | | Complement factor I |
| CH3L1_MOUSE | | Chitinase-3-like protein 1 |
| CH3L3_MOUSE | | Chitinase-3-like protein 3 |
| CH3L4_MOUSE | | Chitinase-3-like protein 4 |
| CHIA_MOUSE | | Acidic mammalian chitinase |
| CLCA1_MOUSE | | Calcium-activated chloride channel regulator 1 |
| CLIC1_MOUSE | | Chloride intracellular channel protein 1 |
| CLUS_MOUSE | | Clusterin |
| CO3_MOUSE | | Complement C3 |
| CO5_MOUSE | | Complement C5 |
| COF1_MOUSE | | Cofilin-1 |
| COQ6_MOUSE | | Ubiquinone biosynthesis monooxygenase COQ6 |
| CP2CT_MOUSE | | Cytochrome P450 2C29 |
| CP2F2_MOUSE | | Cytochrome P450 2F2 |
| CP4CA_MOUSE | | Cytochrome P450 4A12A |
| CRAMP_MOUSE | | Cathelin-related antimicrobial peptide |
| CRIP2_MOUSE | | Cysteine-rich protein 2 |
| CSF2_MOUSE | | Granulocyte-macrophage colony-stimulating factor |
| CSRP1_MOUSE | | Cysteine and glycine-rich protein 1 |
| CXL15_MOUSE | | C-X-C motif chemokine 15 |
| CYTC_MOUSE | | Cystatin-C |
| DEST_MOUSE | | Destrin |
| DPYL2_MOUSE | | Dihydropyrimidinase-related protein 2 2 |
| ECM1_MOUSE | | Extracellular matrix protein 1 |
| ECP1_MOUSE | | Eosinophil cationic protein 1 |
| ECP2_MOUSE | | Eosinophil cationic protein 2 |
| EF1A1_MOUSE | | Elongation factor 1-alpha 1 |
| EF2_MOUSE | | Elongation factor 2 |
| ENOA_MOUSE | | Alpha enolase |
| ENOB_MOUSE | | Beta enolase |
| ESTN_MOUSE | | Liver carboxylesterase N |
| EZRI_MOUSE | | Ezrin |
| FETUA_MOUSE | | Alpha-2-HS-glycoprotein Fetuin A |
| FETUB_MOUSE | | Fetuin B |
| FHL1_MOUSE | | Four and a half LIM domains protein 1 |
| FINC_MOUSE | | Fibronectin |
| FGF1_MOUSE | | Heparin-binding growth factor 1 |
| G3P_MOUSE | | Glyceraldehyde-3-phosphate dehydrogenase |
| GDIR1_MOUSE | | Rho GDP-dissociation inhibitor 1 |
| GELS_MOUSE | | Gelsolin |
| GLRX1_MOUSE | | Glutaredoxin-1 |
| GPX3_MOUSE | | Glutathione peroxidase 3 |
| GRP78_MOUSE | | 78 kDa glucose-regulated protein |
| GSDC2_MOUSE | | Gasdermin-C2 |
| GSTA3_MOUSE | | Glutathione S-transferase A3 |
| GSTA4_MOUSE | | Glutathione S-transferase A4 |
| GSTM1_MOUSE | | Glutathione S-transferase Mu 1 |
| GSTM2_MOUSE | | Glutathione S-transferase Mu 2 |
| GSTO1_MOUSE | | Glutathione S-transferase omega-1 |
| GSTP1_MOUSE | | Glutathione S-transferase P 1 |
| H11_MOUSE | | Histone H1.1 |
| H2A1F_MOUSE | | Histone H2A type 1-F |
| H2B1A_MOUSE | | Histone H2B type 1-A |
| H2B1B_MOUSE | | Histone H2B type 1-B |
| H4_MOUSE | | Histone H4 |
| HBA_MOUSE | | Hemoglobin A |
| HBB1_MOUSE | | Hemoglobin B1 |
| HBB2_MOUSE | | Hemoglobin B2 |
| HBE_MOUSE | | Hemoglobin E |
| HEMO_MOUSE | | Hemopexin |
| HINT1_MOUSE | | Histidine triad nucleotide-binding protein 1 |
| HMGB1_MOUSE | | High mobility group protein B1 |
| HOP_MOUSE | | Homeodomain-only protein |
| HPT_MOUSE | | Haptoglobin |
| HS90A_MOUSE | | Heat shock protein HSP 90-alpha |
| HS90B_MOUSE | | Heat shock protein HSP 90-beta |
| HSP72_MOUSE | | Heat shock-related 70 kDa protein 2 |
| HSP7C_MOUSE | | Heat shock cognate 71 kDa protein |
| HVM12_MOUSE | | Ig heavy chain V region MOPC 104E |
| HVM27_MOUSE | | Ig heavy chain V-III region A4 |
| HVM36_MOUSE | | Ig heavy chain V region 441 |
| ICAM1_MOUSE | | Intercellular adhesion molecule 1 |
| IDHC_MOUSE | | Isocitrate dehydrogenase [NADP] cytoplasmic |
| IGH1M_MOUSE | | Ig gamma-1 chain C region, membrane-bound form |
| IGHA_MOUSE | | Ig alpha chain C region |
| IGHG3_MOUSE | | Ig gamma-3 chain C region |
| IGHM_MOUSE | | Ig mu chain C region secreted form |
| IGJ_MOUSE | | Immunoglobulin J chain |
| IGKC_MOUSE | | Ig kappa chain C region |
| INMT_MOUSE | | Indolethylamine N-methyltransferase |
| ITIH2_MOUSE | | Inter-alpha-trypsin inhibitor heavy chain H2 |
| ITIH3_MOUSE | | Inter-alpha-trypsin inhibitor heavy chain H3 |
| K2C1_MOUSE | | Keratin, type II cytoskeletal 1 |
| K2C73_MOUSE | | Keratin, type II cytoskeletal 73 |
| KCRB_MOUSE | | Creatine kinase B-type |
| KNG1_MOUSE | | Kininogen 1 |
| KPYM_MOUSE | | Pyruvate kinase isozymes M1/M2 |
| KV2A7_MOUSE | | Ig kappa chain V-II region 26-10 |
| KV3A1_MOUSE | | Ig kappa chain V-III region PC 2880/PC 1229 |
| LDHA_MOUSE | | L-lactate dehydrogenase A chain |
| LDHB_MOUSE | | L-lactate dehydrogenase B chain |
| LG3BP_MOUSE | | Galectin-3-binding protein |
| LGUL_MOUSE | | Lactoylglutathione lyase |
| LIPL_MOUSE | | Lipoprotein lipase |
| LPLC1_MOUSE | | Long palate, lung and nasal epithelium carcinoma-associated protein 1 |
| LRFN2_MOUSE | | Leucine-rich repeat and fibronectin type-III domain-containing protein 2 |
| LYZ1_MOUSE | | Lysozyme C-1 |
| LYZ2_MOUSE | | Lysozyme C-2 |
| MDHC_MOUSE | | Malate dehydrogenase, cytoplasmic |
| MDHM_MOUSE | | Malate dehydrogenase, mitochondrial |
| MIF_MOUSE | | Macrophage migration inhibitory factor |
| MOES_MOUSE | | Moesin |
| MUG1_MOUSE | | Murinoglobulin-1 |
| MUG2_MOUSE | | Murinoglobulin-2 |
| MYH9_MOUSE | | Myosin-9 |
| MYL6_MOUSE | | Myosin light polypeptide 6 |
| NDKB_MOUSE | | Nucleoside diphosphate kinase B |
| NGAL_MOUSE | | Neutrophil gelatinase-associated lipocalin |
| NUP93_MOUSE | | Nuclear pore complex protein Nup93 |
| OSTP_MOUSE | | Osteopontin |
| PARK7_MOUSE | | Protein DJ-1 |
| PDIA3_MOUSE | | Protein disulfide-isomerase A3 |
| PGAM1_MOUSE | | Phosphoglycerate mutase 1 |
| PGK1_MOUSE | | Phosphoglycerate kinase 1 |
| PGK2_MOUSE | | Phosphoglycerate kinase 2 |
| PGRP1_MOUSE | | Peptidoglycan recognition protein 1 |
| PIGR_MOUSE | | Polymeric immunoglobulin receptor |
| PLMN_MOUSE | | Plasminogen |
| PLSL_MOUSE | | Plastin-2 |
| PLST_MOUSE | | Plastin-3 |
| PPIA_MOUSE | | Peptidyl-prolyl cis-trans isomerase A |
| PRDX1_MOUSE | | Peroxiredoxin-1 |
| PRDX2_MOUSE | | Peroxiredoxin-2 |
| PRDX4_MOUSE | | Peroxiredoxin-4 |
| PRDX5_MOUSE | | Peroxiredoxin-5, mitochondrial |
| PRDX6_MOUSE | | Peroxiredoxin-6 |
| PROF1_MOUSE | | Profilin-1 |
| PROP_MOUSE | | Properdin |
| PRVA_MOUSE | | Parvalbumin alpha |
| PSPB_MOUSE | | Pulmonary surfactant-associated protein B |
| PTMS_MOUSE | | Parathymosin |
| RADI_MOUSE | | Radixin |
| RETNA_MOUSE | | Resistin-like alpha |
| RNAS4_MOUSE | | Ribonuclease 4 |
| RSSA_MOUSE | | 40S ribosomal protein SA |
| S10A8_MOUSE | | Protein S100-A8 |
| S10A9_MOUSE | | Protein S100-A9 |
| S10AB_MOUSE | | Protein S100-A11 |
| S14L2_MOUSE | | SEC14-like protein 2 |
| SAMP_MOUSE | | Serum amyloid P-component |
| SBP1_MOUSE | | Selenium-binding protein 1 |
| SBP2_MOUSE | | Selenium-binding protein 2 |
| SFTPA_MOUSE | | Pulmonary surfactant-associated protein A |
| SFTPD_MOUSE | | Pulmonary surfactant-associated protein D |
| SH3L1_MOUSE | | SH3 domain-binding glutamic acid-rich-like protein |
| SODC_MOUSE | | Superoxide dismutase [Cu-Zn] |
| SODE_MOUSE | | Extracellular superoxide dismutase [Cu-Zn] |
| SPA11_MOUSE | | Serpin A11 |
| SPA3C_MOUSE | | Serine protease inhibitor A3C |
| SPA3K_MOUSE | | Serine protease inhibitor A3K |
| SPA3N_MOUSE | | Serine protease inhibitor A3N |
| SPB6_MOUSE | | Serpin B6 |
| STK31_MOUSE | | Serine/threonine-protein kinase 31 |
| TAGL_MOUSE | | Transgelin |
| TAGL2_MOUSE | | Transgelin-2 |
| TBA1A_MOUSE | | Tubulin alpha-1A chain |
| TBA1B_MOUSE | | Tubulin alpha-1B chain |
| TBB2A_MOUSE | | Tubulin beta-2A chain |
| TBB2C_MOUSE | | Tubulin beta-2C chain |
| TBB3_MOUSE | | Tubulin beta-3 chain |
| TBB5_MOUSE | | Tubulin beta-5 chain |
| TBB6_MOUSE | | Tubulin beta-6 chain |
| TBCA_MOUSE | | Tubulin-specific chaperone A |
| TERA_MOUSE | | Transitional endoplasmic reticulum ATPase |
| TGM2_MOUSE | | Protein-glutamine gamma-glutamyltransferase 2 |
| THIC_MOUSE | | Acetyl-CoA acetyltransferase, cytosolic |
| THRB_MOUSE | | Prothrombin |
| TKT_MOUSE | | Transketolase |
| TPIS_MOUSE | | Triosephosphate isomerase |
| TRFE_MOUSE | | Serotransferrin |
| TRFL_MOUSE | | Lactotransferrin |
| TTHY_MOUSE | | Transthyretin |
| UROM_MOUSE | | Uromodulin |
| UTER_MOUSE | | Uteroglobin |
| VINC_MOUSE | | Vinculin |
| VTDB_MOUSE | | Vitamin D-binding protein OS |
| VTNC_MOUSE | | Vitronectin |
| WFDC2_MOUSE | | WAP four-disulfide core domain protein 2 |
| XPO2_MOUSE | | Exportin-2 |
| ZA2G_MOUSE | | Zinc-alpha-2-glycoprotein |

All proteins were identified at 95% significance level with at least 2 peptides. Accession Uniprot knowledgebase v.56 www.uniprot.org
