# Supplementary material for: Comprehensive multiplexed protein quantitation delineates eosinophilic and neutrophilic experimental asthma
Source: BMC Pulm Med. 2014 Jul 4;14:110. doi: 10.1186/1471-2466-14-110 (PMC4137270; doi:10.1186/1471-2466-14-110)
Supplement: Additional file 2: Figure S1 — Protein changes as detected by means of mass spectrometry based proteomics. Statistical significance (p < 0.05) is indicated with * OVA/LPS vs C; # OVA/LPS vs OVA/OVA; % OVA/LPS vs OVA/LPS/GC and &OVA/OVA vs C. Figure S2. Protein changes as detected by means of Bio-Plex analysis. Statistical significance (p < 0.05) is indicated with * OVA/LPS vs C; # OVA/LPS vs OVA/OVA; % OVA/LPS vs OVA/LPS/GC and &OVA/OVA vs C. [file 1471-2466-14-110-S2.pdf]

2. SI Figures

Figure S1: Protein changes as detected by means of mass spectrometry based proteomics. Statistical significance ($p<0.05$) is indicated with * OVA/LPS vs C; # OVA/LPS vs OVA/OVA; % OVA/LPS vs OVA/LPS/GC and &OVA/OVA vs C

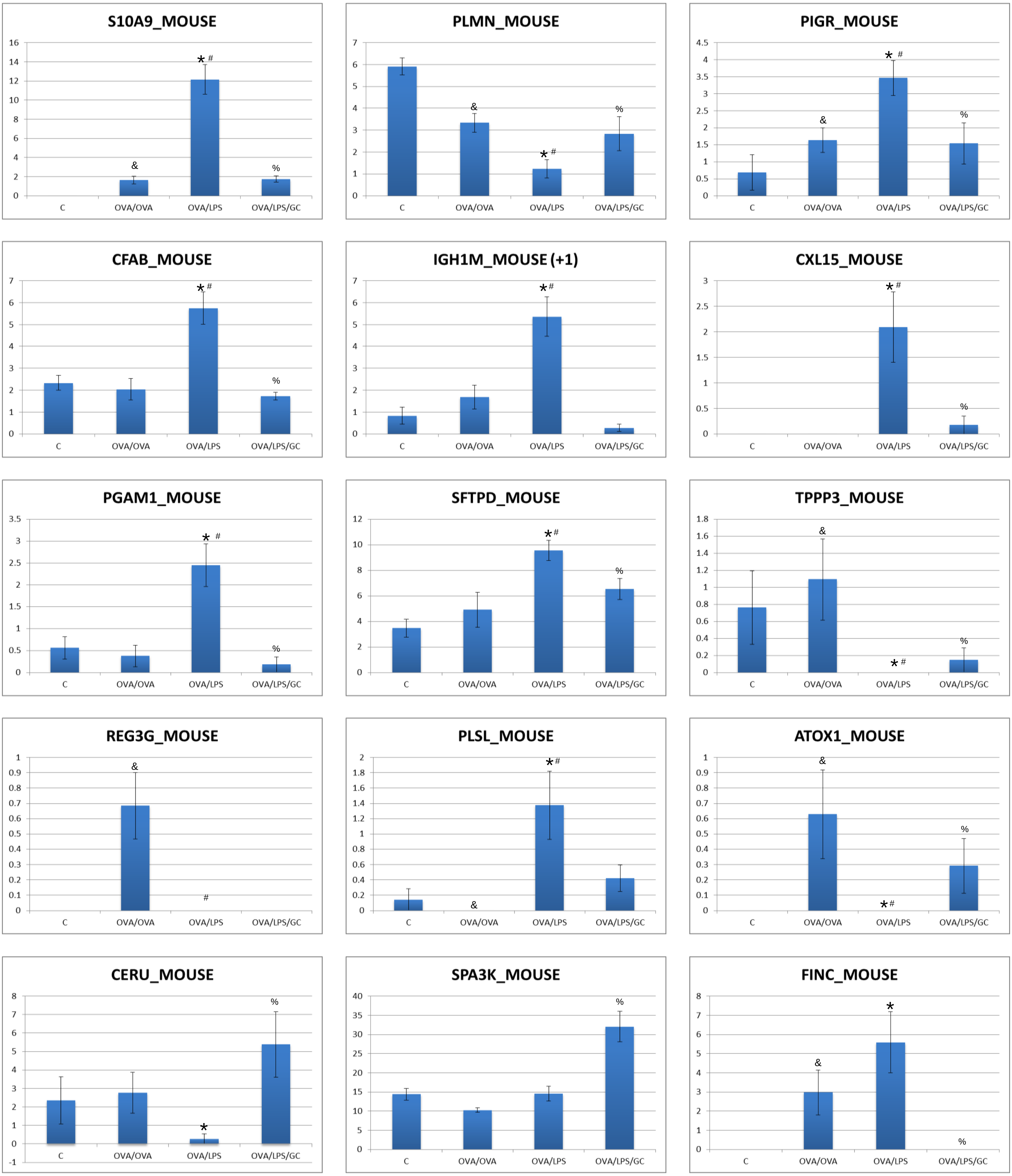

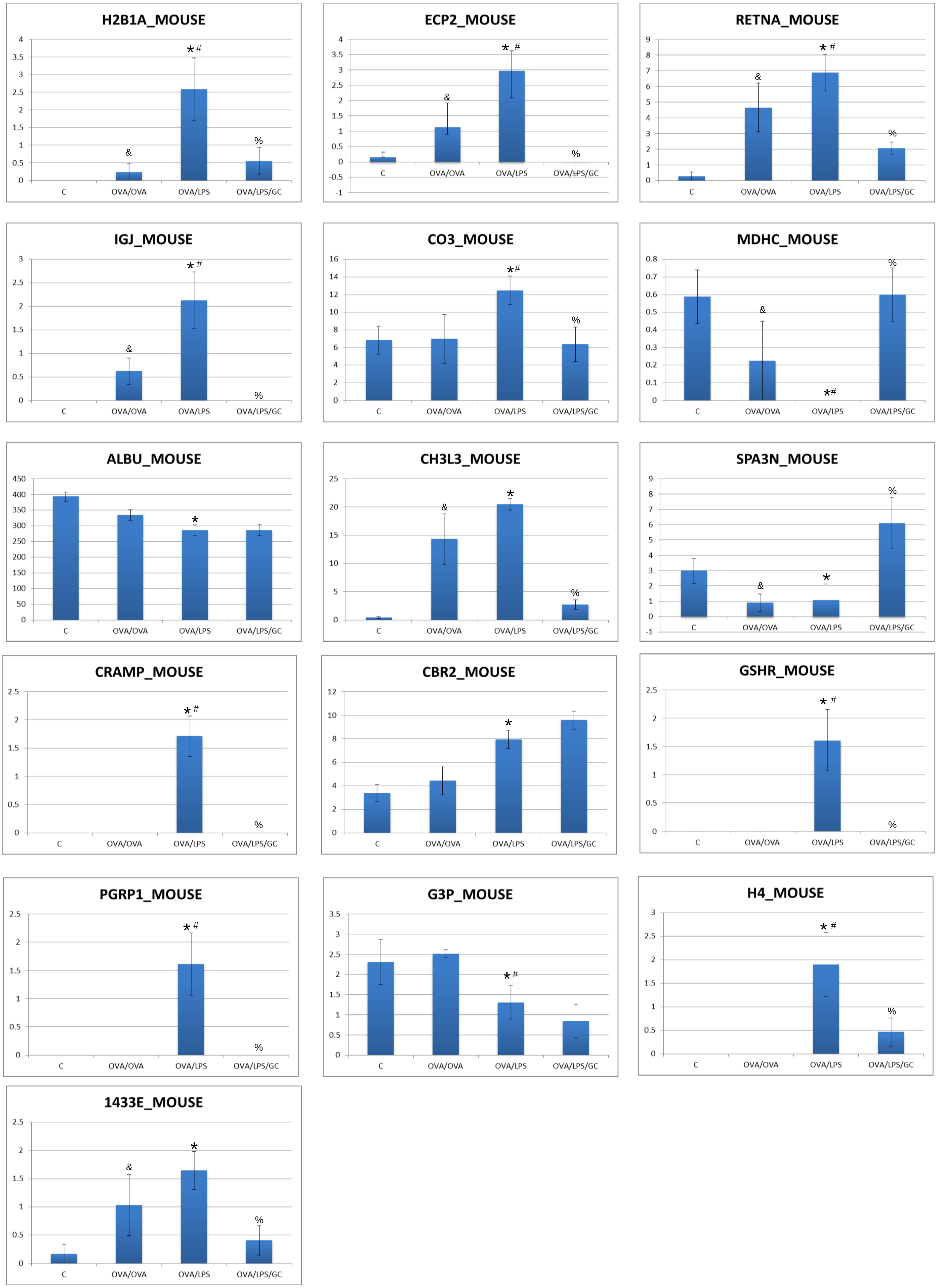

H2B1A_MOUSE
0 0.5 1 1.5 2 2.5 3 3.5 4
C OVA/OVA OVA/LPS OVA/LPS/GC
& *# %
ECP2_MOUSE
-1 -0.5 0 0.5 1 1.5 2 2.5 3 3.5 4
C OVA/OVA OVA/LPS OVA/LPS/GC
& *# %
RETNA_MOUSE
0 1 2 3 4 5 6 7 8 9
C OVA/OVA OVA/LPS OVA/LPS/GC
& *# %
IGJ_MOUSE
0 0.5 1 1.5 2 2.5 3
C OVA/OVA OVA/LPS OVA/LPS/GC
& *# %
CO3_MOUSE
0 2 4 6 8 10 12 14 16
C OVA/OVA OVA/LPS OVA/LPS/GC
*# %
MDHC_MOUSE
0 0.1 0.2 0.3 0.4 0.5 0.6 0.7 0.8
C OVA/OVA OVA/LPS OVA/LPS/GC
& *# %
ALBU_MOUSE
0 50 100 150 200 250 300 350 400 450
C OVA/OVA OVA/LPS OVA/LPS/GC
*
CH3L3_MOUSE
0 5 10 15 20 25
C OVA/OVA OVA/LPS OVA/LPS/GC
& * %
SPA3N_MOUSE
-1 0 1 2 3 4 5 6 7 8 9
C OVA/OVA OVA/LPS OVA/LPS/GC
& * %
CRAMP_MOUSE
0 0.5 1 1.5 2 2.5
C OVA/OVA OVA/LPS OVA/LPS/GC
*# %
CBR2_MOUSE
0 2 4 6 8 10 12
C OVA/OVA OVA/LPS OVA/LPS/GC
*
GSHR_MOUSE
0 0.5 1 1.5 2 2.5
C OVA/OVA OVA/LPS OVA/LPS/GC
*# %
PGRP1_MOUSE
0 0.5 1 1.5 2 2.5
C OVA/OVA OVA/LPS OVA/LPS/GC
*# %
G3P_MOUSE
0 0.5 1 1.5 2 2.5 3 3.5
C OVA/OVA OVA/LPS OVA/LPS/GC
*#
H4_MOUSE
0 0.5 1 1.5 2 2.5 3
C OVA/OVA OVA/LPS OVA/LPS/GC
*# %
1433E_MOUSE
0 0.5 1 1.5 2 2.5
C OVA/OVA OVA/LPS OVA/LPS/GC
& * %

Figure S2: Protein changes as detected by means of Bio-Plex analysis. Statistical significance ($p<0.05$) is indicated with * OVA/LPS vs C; # OVA/LPS vs OVA/OVA; % OVA/LPS vs OVA/LPS/GC and &OVA/OVA vs C

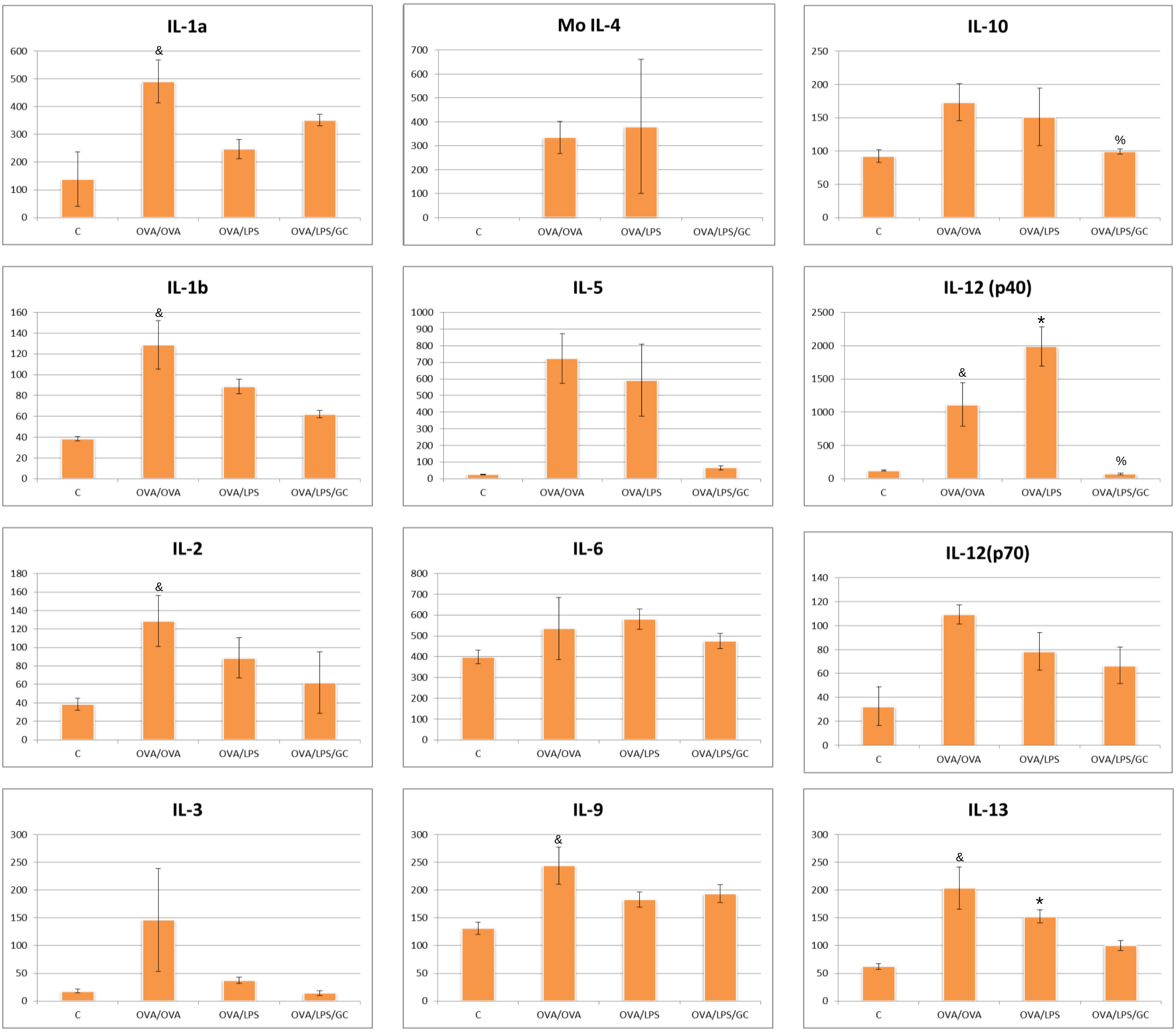

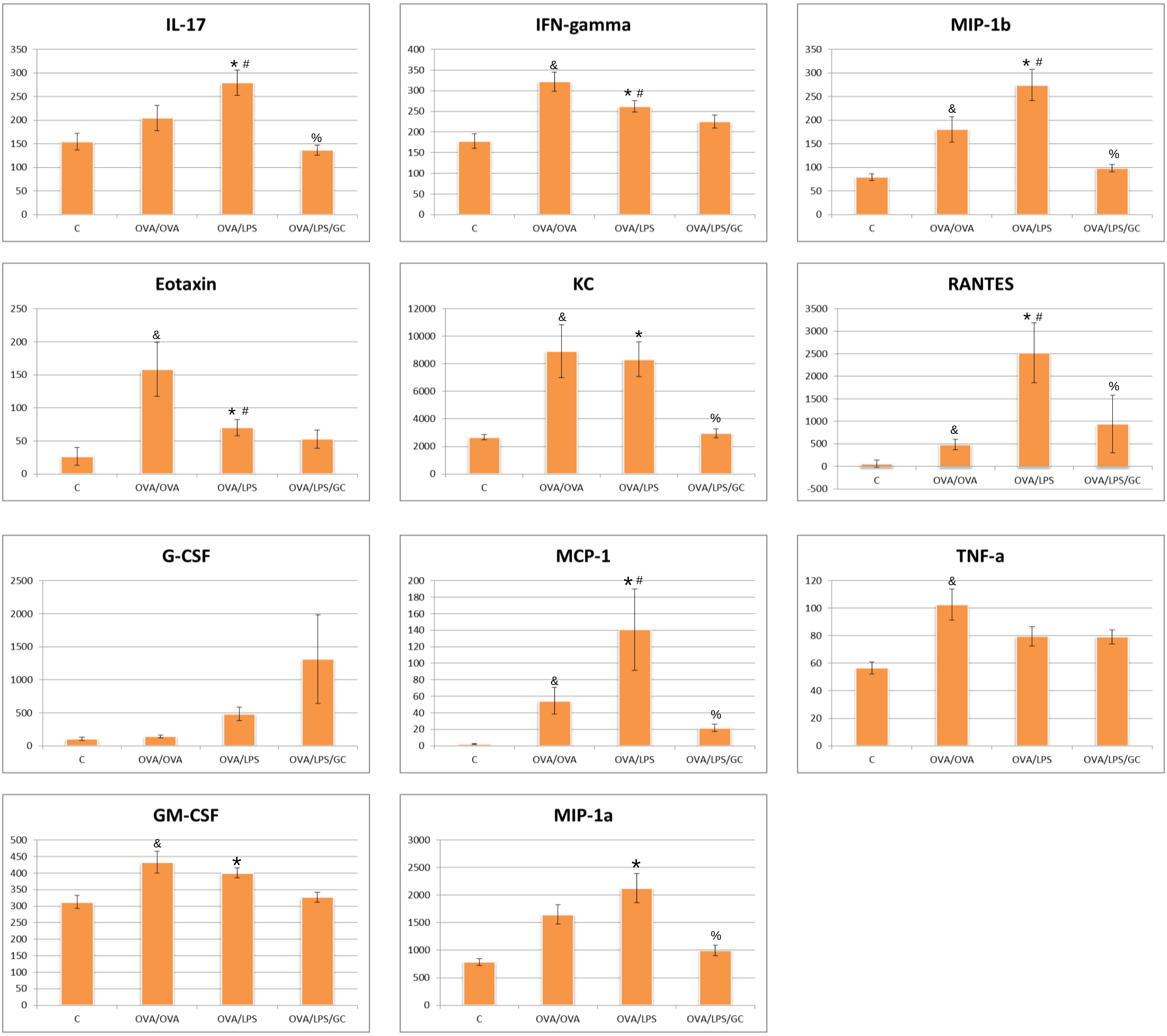

IL-17
350
300
250
200
150
100
50
0
C
OVA/OVA
OVA/LPS
OVA/LPS/GC
* #
%
IFN-gamma
400
350
300
250
200
150
100
50
0
C
OVA/OVA
OVA/LPS
OVA/LPS/GC
&
* #
MIP-1b
350
300
250
200
150
100
50
0
C
OVA/OVA
OVA/LPS
OVA/LPS/GC
&
* #
%
Eotaxin
250
200
150
100
50
0
C
OVA/OVA
OVA/LPS
OVA/LPS/GC
&
* #
KC
12000
10000
8000
6000
4000
2000
0
C
OVA/OVA
OVA/LPS
OVA/LPS/GC
&
*
%
RANTES
3500
3000
2500
2000
1500
1000
500
0
-500
C
OVA/OVA
OVA/LPS
OVA/LPS/GC
&
* #
%
G-CSF
2500
2000
1500
1000
500
0
C
OVA/OVA
OVA/LPS
OVA/LPS/GC
MCP-1
200
180
160
140
120
100
80
60
40
20
0
C
OVA/OVA
OVA/LPS
OVA/LPS/GC
&
* #
%
TNF-a
120
100
80
60
40
20
0
C
OVA/OVA
OVA/LPS
OVA/LPS/GC
&
GM-CSF
500
450
400
350
300
250
200
150
100
50
0
C
OVA/OVA
OVA/LPS
OVA/LPS/GC
&
*
MIP-1a
3000
2500
2000
1500
1000
500
0
C
OVA/OVA
OVA/LPS
OVA/LPS/GC
*
%
